# The Last Mile—Community Engagement and Conditional Incentives to Accelerate Polio Eradication in Pakistan: Study Protocol for a Quasi-Experimental Trial

**DOI:** 10.3390/mps6050083

**Published:** 2023-09-08

**Authors:** Jai K. Das, Amira Khan, Farhana Tabassum, Zahra Ali Padhani, Atif Habib, Mushtaq Mirani, Abdu R. Rahman, Zahid Ali Khan, Arjumand Rizvi, Imran Ahmed, Zulfiqar Bhutta

**Affiliations:** 1 Institute for Global Health and Development, Aga Khan University, Karachi 74800, Pakistan; farhana.tabassum@aku.edu (F.T.); mushtaque.mirani@aku.edu (M.M.); rahman.ramzan@aku.edu (A.R.R.); zahidali.khan@aku.edu (Z.A.K.); zulfiqar.bhutta@aku.edu (Z.B.); 2 Division of Women and Child Health, Aga Khan University, Karachi 74800, Pakistan; 3 Centre for Global Child Health, The Hospital for Sick Children, Toronto, ON M5G 1X8, Canada; amira.khan@sickkids.ca; 4 Robinson Research Institute, University of Adelaide, Adelaide, SA 5000, Australia; zahraali.padhani@adelaide.edu.au; 5 Centre of Excellence in Women and Child Health, Aga Khan University, Karachi 74800, Pakistan; habibatif@yahoo.com (A.H.); arjumand.rizvi@aku.edu (A.R.); imran.ahmed@aku.edu (I.A.)

**Keywords:** incentives, community mobilization, polio, Pakistan, immunization, refusals

## Abstract

Poliomyelitis is a condition of great concern and is endemic in only two countries of the world: Pakistan and Afghanistan. Community mobilization plays a vital role in raising awareness and can help reduce polio vaccine refusals. The objective of this study will be to decrease polio vaccine refusals and zero-dose vaccines by motivating behavior change through the provision of conditional–collective–community-based incentives (C3Is) based on a reduction in polio vaccine refusals. The project will adopt a pretest/post-test quasi-experimental design with two intervention high-risk union councils (HRUCs) and two control union councils (UCs) of peri-urban (Karachi) and rural (Bannu) settings in Pakistan. A participatory community engagement and demand creation strategy with trust-building community mobilization with C3Is, to reduce vaccine refusals and improve polio immunization coverage in two HRUCs, will be used. These UCs will be divided into clusters based on the polio program framework and community groups will be formed in each cluster. These community groups will carry out awareness activities and will be given serial targets to reduce vaccine refusals and those who qualify will be provided C3Is. The project intends to create a replicable model that the government can integrate within health systems for long-term sustainability until the goal of eradication of poliovirus is achieved. The evaluation will be carried out by an independent data collection and analysis team at baseline and endline (after 12 months of intervention). The trial is registered with linicalTrials.gov with number NCT05721274.

## 1. Introduction

Immunization is one of the most successful and cost-effective public health strategies for minimizing numerous infectious diseases, as they stimulate the individual’s own immune system to provide protection against subsequent infections or diseases [1,2]. Vaccines have eradicated illnesses that have affected children, such as polio, from the whole globe, except in a few countries because of a lack of awareness and misconceptions [3,4].

Immunizing children against disease is especially important in the first five years of life [5]. However, the targets of global coverage of childhood vaccines remain distant as the global coverage of childhood vaccinations dropped from 86% in 2019 to 81% in 2021, largely because of the COVID-19 pandemic and associated disruptions [6]. Nonetheless, the coverage of routine childhood vaccination in low-income and middle-income countries (LMICs) remains low or stagnant. An estimated 19.7 million infants did not receive routine immunizations in the year 2021 and around 60% of these children live in just ten LMICs including Pakistan, Ethiopia, India and Nigeria [6]. The Pakistan demographic and health survey (PDHS) showed that the vaccine coverage rate increased gradually from 35% in 1990 to 47% in 2006–2007, 54% in 2012–2013 and 66% in 2017–2018 [7]. However, more efforts are needed to achieve universal immunization coverage.

Despite extensive eradication initiatives, poliovirus has not been eliminated from the world’s population, and Pakistan and Afghanistan are the only two countries where wild poliovirus (WPV) is still present. The reported cases from Pakistan though have declined significantly over the years but are still being reported: 12 polio cases in 2018, 147 in 2019, 84 in 2020, 1 in 2021 and 20 in 2022. Reference [8] although there has been a substantial decrease in reported polio cases in Pakistan, there is still some ongoing transmission of the virus. This implies that despite efforts to control and eliminate polio, the disease has not been completely eradicated in the country and remains a concern as vaccination rates are not universal. In 1978, Pakistan initiated the extended program on immunization (EPI) to immunize all children aged 0 to 23 months against twelve vaccine-preventable illnesses. These services are provided for free by the public health delivery network, which includes fixed centers and outreach programs coordinated by vaccinators with the assistance of lady health workers (LHW).

Despite rigorous efforts, misconceptions and misunderstandings remain regarding childhood immunizations, which lead to vaccine refusals [9]. People connect polio vaccines with infertility and hence are afraid of the consequences [4,10], and community decisions are mostly influenced by their faith and perceptions regarding the safety and efficacy of vaccines [11]. The major barriers to vaccination uptake are also a lack of appropriate information about these issues due to poor or inadequate communication and a lack of knowledge that negatively affect vaccination rates and awareness [10]. Pakistan is at risk of being the last active reservoir of poliomyelitis virus in the entire world. Although LHWs are highly admired because they have been recognized in the community for their abilities to deliver primary care and preventative services for the health of mother and child, they are only aware of the generic reason for refusals rather than real-life traumatic experiences, which are still unanswered and need to be explored to eradicate polio disease [10]. Vaccine confidence plays a vital role in the acceptance and refusals of the vaccination program [12], and thus improving communication about vaccination can be a crucial factor in enhancing vaccination outcomes [10,13].

Refusals are often linked to “polio fatigue”, where communities deprived of many basic services, i.e., health, nutrition, water and sanitation, grow weary of repeated knocks at the door for polio activities. And the spread of misinformation and propaganda, fueled by social media, also scales up the mistrust in the polio vaccine, which has now materialized as real community resistance to vaccination [14]. The “public” in public health implies an important link between people and health systems. Research has identified that community belonging and community influence can be central to behavior change at the individual level [15,16]. Hence, external influences such as incentives can act as “cues to action” to motivate caregivers to change their behavior [17,18]. This study aims to evaluate the impact of a novel community engagement strategy with a conditional–collective–community-based incentive (C3I) on reducing polio vaccine refusals in the high-risk union councils (HRUCs) of Pakistan. The incentives proposed are novel as they are conditional on the pretext of collective improvement in community behavior rather than individual behavior, which has never been tested in the context of reducing polio vaccine refusals.

## 2. Conceptual Framework

**Figure 1:**
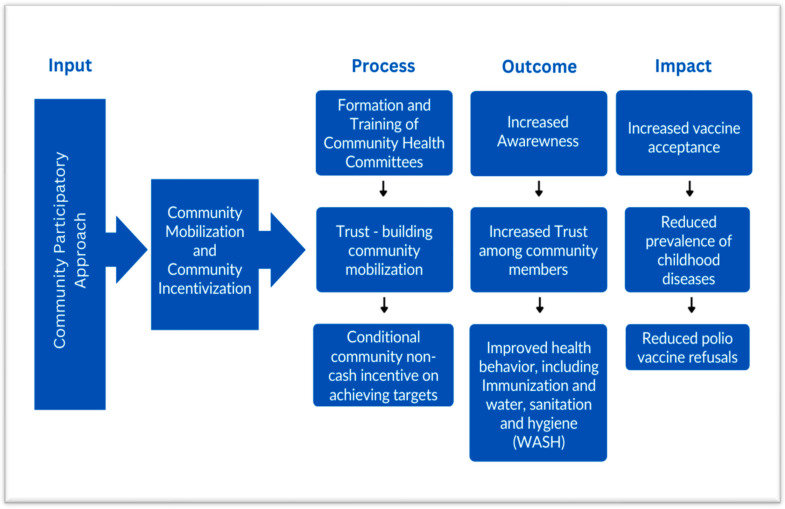
The proposed theory of change for reducing polio vaccine refusals.

The use of resourceful incentives to promote optimal health behavior is a critical intersection of behavioral science and public health. Therefore, the Health Belief Model is adapted for this project with some modifications (Figure 1). This model assists in understanding beliefs that lead to behavior intentions and consequent behaviors. It also helps to unpack how subjective norms, attitudes towards the behavior and perceived behavioral control leads to a behavior intention [19]. A part of this model highlights factors that can persuade individuals to adopt behavioral changes and external influences such as incentives that can act as “cues to action” to motivate caregivers to change their behavior [17,18]. Providing incentives can improve the resource-poor environments of disadvantaged communities, which can help to promote optimal health behaviors such as vaccination [20]. It is also known that a group strategy that is beneficial and unifying and of which the whole community has ownership can be more effective in convincing individual community members, as community belonging and community-level influence can be central to behavior change at the individual level [15,16].

## 3. Methodology

### 3.1. Objective

The objective of this study is to decrease polio vaccine refusals and zero-dose polio vaccines by motivating behavior change through community mobilization and C3Is.

### 3.2. Study Design

The study will adapt a pre/post-test quasi-experimental design with two intervention HRUCs and two control HRUCs. For each targeted intervention HRUC, a separate matched control HRUC of comparable size and location will be included. Union councils are the lowest administrative unit in Pakistan.

### 3.3. Intervention

The intervention involves engaging and empowering community groups. These groups will be formed in each of the intervention high-risk union councils (HRUCs), who will then engage in community mobilization activities to improve polio vaccine uptake in their respective clusters. The design of this trial is based on the previous trial which was conducted in a rural setting of Pakistan and aimed at improving routine childhood vaccine coverage amongst other outcomes [21]. The implementation of this study will be guided by the findings and lessons learned from the previously mentioned trial. All the selected UCs are declared as polio high risk by the World Health Organization (WHO) in response to persistent virus transmission and were chosen in consultation with the national and provincial Emergency Operation Cell (EOC), which is a multi-sectoral body looking at polio in Pakistan.

The target of this Intervention is to reduce polio vaccine refusals and increase polio vaccine coverage in the target high-risk areas, especially for the last remaining resistant populations to help advance the global agenda of polio eradication. The Institute for Global Health and Development at Aga Khan University (AKU), Karachi, together with the Trust for Vaccines and Immunization (TVI) led this project in close collaboration with district, provincial and federal government. The project intends to create a replicable model that the government can integrate within health systems for long-term sustainability until the goal of eradication of poliovirus is achieved.

### 3.4. Study Setting

The study will be conducted in two intervention and two control HRUCs. There will be one intervention HRUC and one control HRUC, each from a peri-urban district of Karachi and a rural district of Banu, Pakistan. The intervention HRUC of Karachi is Haji Mureed Goth and the control is UC 49 in Nazimabad in the central district, which has an area of 69 km^2^, a population of approximately 2,971,626 and a density of 43,000/km^2^ and is divided into four towns [22]. While the intervention HRUC of Bannu city is Mira Khel and the control is Khwajamad, both of these areas belong to Bannu district, which has a population of 1,210,183 and a density of 610/km^2^ with four tehsils [23]. Both control and intervention UCs were chosen in consultation with the EOC as they have similar demographics and vaccine coverage rates.

### 3.5. Study Duration and Registration

The intervention will be delivered over a period of one year and the trial is registered on clinicaltrial.gov with trial registration number NCT03594279 [24]

### 3.6. Outcomes

The outcomes of this trial will be polio vaccine refusals both in persistently missed children (PMC) and still missed children (SMC). The government conducts serial house-to-house polio vaccine campaigns and children who miss one campaign are labelled as SMC, while children who consistently miss the polio vaccine for three consecutive rounds are labelled as PMC.

### 3.7. Study Population

The population of interest for this study is all households with at least one child under the age of five years and permanent residents of the selected HRUCs.

### 3.8. Sample Size and Sampling Strategy

**Figure 2:**
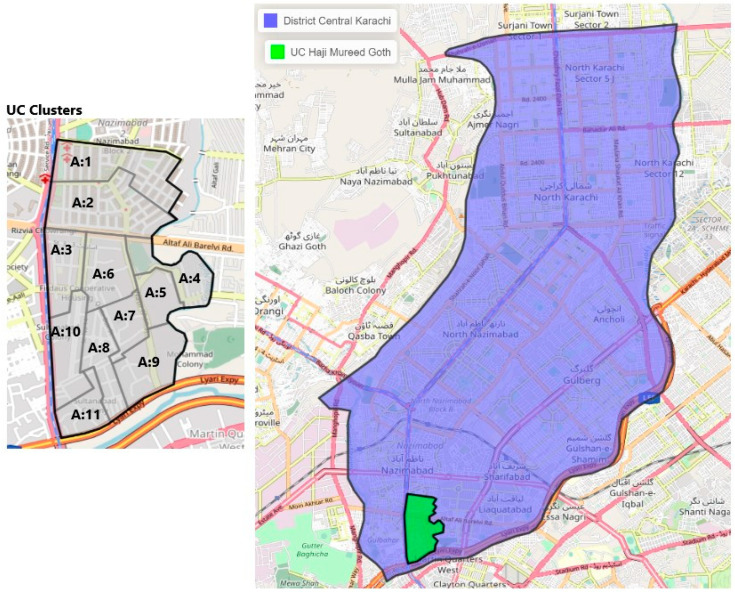
Map of selected district and HRUCs and the intervention clusters (Karachi, Haji Mureed Goth).

**Figure 3:**
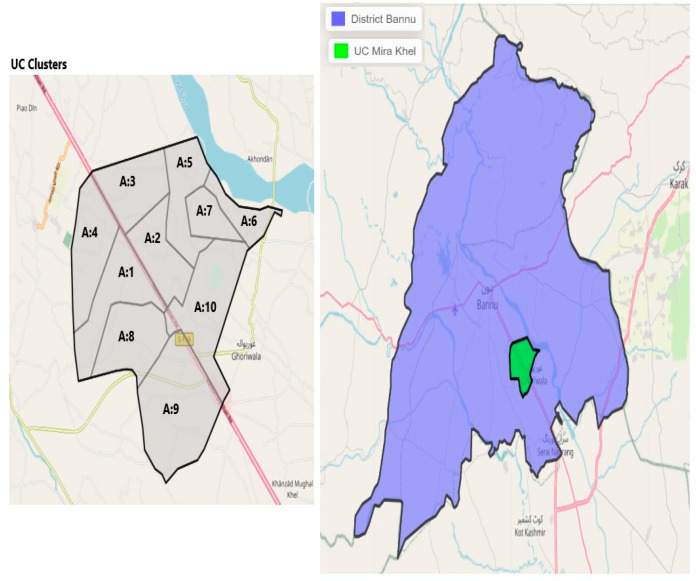
Map of selected district and HRUCs and the intervention clusters (Bannu, Mira Khel).

The polio program is well established, and demarcated vaccination areas are assigned to each vaccination team. Therefore, the areas allotted to the vaccination team will be considered as one cluster in the proposed study. The first intervention HRUC (Haji Mureed Goth) is divided into four wards, distributed into 11 areas that are each supervised by “area supervisors”, and has a total of 47 community health workers (CHWs) from the Polio Eradication Program, hence we will form 11 clusters in this HRUC. The second intervention HRUC (Mira-Khel) of district Bannu is divided into 10 areas, which are supervised by 10 area supervisors, and has a total of 43 polio LHWs from the Polio Eradication Program, thus 10 clusters will be formed in Bannu (Figure 2 and Figure 3).

## 4. Project Implementation

**Figure 4:**
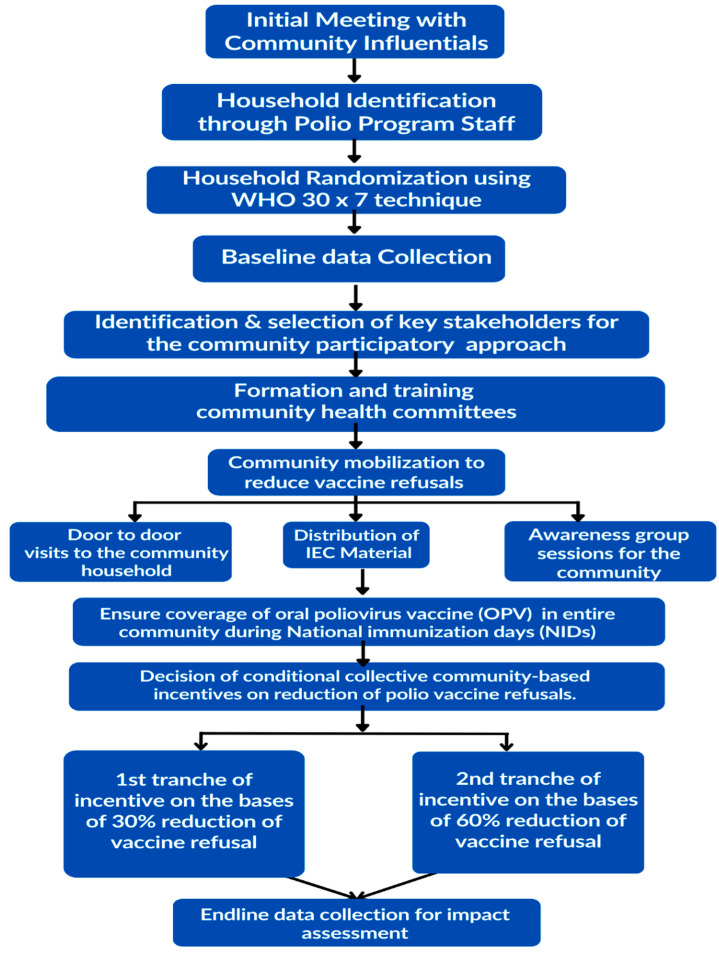
Study flow chart.

The project implementation will be divided into three phases: the planning phase, the implementation phase and the close-out phase (Figure 4).

### 4.1. Planning Phase

The project intends to build on previous experiences and engage primarily with local stakeholders, community volunteers and influencers. However, the engagement of local health authorities and workers will be integral to the project. The initial planning phase will focus on the identification of and consultations with stakeholders, obtaining relevant permissions and approvals (ethical and regulatory) to ensure survey and mobilization activities can be implemented smoothly.

This study will adopt a conditional incentivization approach, preceded by trust-building community mobilization (Figure 1). Clusters will be formed based on EOC/WHO distributed areas based on area supervisors. Community groups will be formed separately for male and female members in each of the 21 clusters in the two intervention HRUCs and they will be responsible for carrying out community mobilization. The community groups will comprise 6–8 members, including UC members, local elders/elites, religious leaders and prominent male and female members. Each cluster may contain more than one mohalla/area/village of the union council and ensure the representations of all segments (caste, ethnicity and religious sects of the community). The community groups will undergo a comprehensive 2–3-day training on specific messages regarding the polio vaccination to assist them in planning and carrying out awareness and motivational activities.

Simple, pictorial and easy-to-understand information education communication (IEC) material has been specifically designed for this project to sensitize the community about the importance of childhood diseases, vaccine-preventable diseases and how repeated polio rounds are important for their children. The IEC material will be distributed during community mobilization sessions with an emphasis on the key messages. The community groups, with the support of the project team, will conduct awareness sessions about the effects of the disease and the importance of vaccines and distribute IEC materials among the participants at a community level. IEC materials play a crucial role in promoting vaccine acceptance and addressing vaccine hesitancy [25,26,27]. They provide accurate and accessible information about vaccines, their benefits, vaccine schedule and potential risks. It was ensured that our IEC aligned with national guidelines.

### 4.2. Implementation Phase

#### 4.2.1. Community Mobilization

The committees will facilitate two-hour group meetings to be held every week in every village/mohalla of their catchment area and focus on issues mentioned. The committees will ensure that sessions are held throughout the UC and not concentrated in certain localities. Group activities will also identify community-level health, nutrition and sanitation problems, and focus on finding locally feasible strategies to address them.

These mobilization activities will have a cross-cutting theme of community engagement and empowerment where the community is central to the mobilization process and in planning the sessions and involved with the committees in different capacities to aid the process. These community sessions would be helpful to identify parents’/the community’s underlying fears and socio-psychological factors related to the vaccines such as painful experiences, mishandling, doubts, fear of vaccine-related side effects, mistrust, etc., and eventually to improve the acceptance of the vaccine to reduce refusals and missed children in the following NID campaign. All project activities will be planned to keep in mind the cultural sensitivity, norms and values of the respective area. Refusals and missed children’s data will be collected from the polio supervisors along with their major reasons for denial after each national immunization day (NID) campaign and will be reviewed thoroughly to identify the number of refusals and missed children in each cluster. The community groups will be responsible for arranging and conducting monthly awareness sessions and visiting door to door, focusing on polio refusals and missed children during the previous campaigns in each intervention cluster.

To build trust in the health and vaccination system the committees will engage LHWs, private practitioners, local health officials, health workers and vaccinators to participate. These will be opportunities to address fears, concerns and suspicions of the community members regarding polio vaccination. In addition, this will help to build a relationship of respect and trust with health workers, which will help increase receptivity to the polio vaccination.

#### 4.2.2. Conditional–Collective–Community-Based Incentives (C3I)

The clusters which are successful in reducing vaccine refusals (SMC and PMC) will be eligible to receive incentives. The C3Is will be conditioned on improvements in vaccine acceptance and will not target individuals or be based on individual practices; rather, they will be targeted to benefit the entire community. These non-cash, need-based incentives will be decided with the consensus of community groups and aim to improve infrastructure linked to health, including drinking water and sanitation facilities, at the community level. The total cost of these incentives will be shared by the project (75%) and the community (25%) to improve ownership. The model of decision and delivery is the same as the CoMIC trial [21].

C3Is will be available for each cluster (21 clusters in two HRUCs) in the intervention clusters, provided they reduce refusals and hence increase the OPV and IPV coverage in a pre-defined period based on the usual data collected in the routine polio vaccine campaigns. We will calculate the mean SMC and PMC in the preceding three campaigns in each cluster, and to be eligible to receive incentives in two phases, each cluster will have to achieve the following targets over a one-year period:

At 5 months—30% improvement (from baseline)—30% reduction in SMC and PMC in the next two campaigns.

At 12 months—50% improvement (from baseline)—50% reduction in SMC and PMC in the last two campaigns.

These non-cash, need-based incentives will be decided with the consensus of UC committees and aim to improve infrastructure linked to health, including but not limited to water supply facilities, toilets and sanitation facilities. This unique incentive scheme will not target individuals or be based on individual practices; rather, it will benefit the community, as we will look at the improvement in the coverage of the defined cluster. Providing incentives that can improve the resource-poor environments of disadvantaged communities can help promote optimal health behaviors such as vaccination [20].

Community belonging and community-level influence can be central to behavior change at the individual level [15,20]. This strategy is formulated on the assumption that a group strategy that is beneficial, unifying and of which the whole community has ownership can be more effective in convincing individual community members.

### 4.3. Close-Out Phase

The close-out phase will be dedicated to the delivery of incentives, an end-line survey, data compilation, evaluation and report writing. This will be followed by the dissemination of results and discussion with local and provincial government departments and stakeholders.

## 5. Monitoring and Evaluations

### 5.1. Team Composition

The project-based team will be hired consisting of two teams—one for each intervention area. Thus, four female and two male staff will be engaged in field sites. They will be responsible for data collection, identification and meeting with stakeholders, committee formation, community mobili30zation, etc. These activities will be carried out under the supervision of one coordinator/manager separately for each study site. Mostly Sindhi, Urdu and Pashto communities are residing in the Haji Mureed Goth intervention UC at Karachi central district, whereas in the Mira-Khel UC of Bannu district there is only a Pashto community, which is why the project teams will be hired according to the above ethnicities to roll out the entire mobilization initiative and support Community Resource Persons (CRPs) and polio program workers.

### 5.2. Data Collection

The performance and refusal rates will be assessed via independent and robust monitoring through independent surveys, data from the National Emergency Operations Centre (NEOC) and PPEP data for OPV coverage.

Apart from the regular data of NEOC, the project team will also collect data at baseline and endline. Two-stage cluster sampling will be adopted for the baseline and endline. The union councils (UC) will be considered as independent strata and each UC will be subdivided into 30 equal areas (clusters), the WHO 30 × 7 technique will be implemented to achieve the sample size and seven households (HHs) will be selected randomly within each cluster using an available sampling frame of the area [28]. Thus, a total of 840 interviews will be conducted, 210 from each of the four areas (two intervention and two control).

Face-to-face interviews will be conducted with eligible participants, after informed written consent is obtained, using a structured data collection instrument which will be based on household information, socio-demographics of the HHs, immunization status and polio campaign. After instrument development and before starting field activities, a pilot survey will be conducted; this exercise will be carried out in all non-targeted areas. All steps of the survey will be followed in the pilot exercise including the methodology, instruments, guidelines, etc. Revisions will be made based on the lessons learned in the pilot survey.

### 5.3. Quality Assurance

All the data will be collected electronically, and the data collection application will be developed in the local language Urdu. Handheld devices running the Android operating system will be used. Java will be used to develop a customized data collection application. MySQL databases will be used at the backend for data storage. This is a popular relational database system that provides efficient storage, retrieval and management of structured data. The data will be exported to statistical software for analysis. The application will have built-in data quality checking features to identify any missing information, inconsistencies, outliers or other errors in real time. The user will be prompted for any such errors at the time of data collection to ensure quality data. Additional validation checks will be performed at the central data repository. Data will be transferred from each handheld device at the end of the day. An institutional server will be used as per IT policy for data hosting and archiving; therefore, data will be transmitted directly to the AKU server via the internet.

In the case that internet is not available at remote locations, the team leaders will manually export a copy of the data to a memory stick to avoid any data loss.

The data collection application will be password-protected. Once a record is saved, it will not be editable by the collection staff. Data stored on handheld devices, as well as during data transfer, will be encrypted and anonymized to avoid any breach of confidentiality of participants’ personal information. Data will be archived and will be stored in a data repository at the AKU. Access to the data repository will be limited to only data management personnel directly involved in the project through their AKU-LAN identification. Once the data are transferred to the main server at AKU, it will be accessible only to the data management personnel involved in the survey. The level of access to the data will be different according to the project role of the person. The data synchronized at the AKU data repository will be replicated to a remote location as backup. The fail-over/slave server will also be maintained for restoration of the database in the event of a disaster that destroys the primary database server. A data collection application installation guide, user manual and database documentation will be created. The database documentation will include field names for all variables, their type, description, codes and value labels. Steps will be defined to transfer these data into SPSS/STATA format, retaining the variable labels and value labels.

### 5.4. Monitoring

The project activities will be regularly and rigorously monitored by the regional managers, who will be specifically trained to supervise this task. The following steps will be ensured during monitoring and quality control in the field:

- Each data collector will be expected to submit/sync only completed and accurate questionnaires, and every day the supervisor will check the data for completeness and timely syncing.
- Any discrepancies or missing data found will be resolved through discussions with the interviewers, reviewing photographs of the vaccination card (if available) or revisiting the HHs if necessary. Steps to improve the next day’s work will also be deliberated upon.
- District supervisors will conduct a validation process to ensure the accuracy and completeness of the collected data. They will specifically focus on verifying the boundaries of households, clusters or segments. The purpose of this validation is to ensure that field workers do not inadvertently or deliberately omit eligible households from the data collection process. By conducting this validation, the district supervisors aim to maintain the quality and integrity of the data.
- A dedicated quality control associate at the data management unit (DMU) will also review pictures of immunization cards which will be taken by survey teams and compare them with the results of the survey to validate the quality of data transcription by data collectors.
- The CHCs’ performance and refusal rates will be assessed via independent and robust monitoring through independent surveys, data from the National Emergency Operations Centre (NEOC) and Polio Eradication Program data for OPV coverage.
- To improve the acceptance of the vaccine along with staff performance, stakeholders’ engagement, the performance of community groups, community mobilization sessions and the frequency of individual and group meetings will be monitored.
- Conditional non-cash need-based incentives will be decided based on reductions in the polio vaccine refusals, PMC and SMC.

## 6. Statistical Analysis

The baseline characteristics of the study population will be reported as frequencies and percentages for categorical variables, and mean, standard deviation (SD) and median interquartile range (IQR) for continuous variables as appropriate. The immunization coverage will be reported overall and according to time and intervention groups. Subgroups including age, gender, maternal years of education, socio-economic status and area of residence will also be compared for immunization coverage. A difference-in-difference (DID) method will be used to estimate the impact of intervention over time. Multivariate analysis will be performed using binary logistic regression at endline to determine the impact of intervention controlling for multiple confounding factors. The analysis will be carried out in STATA version 17 [29].

## 7. Discussion

Immunization has a significant health benefit, particularly for children, but there are many misconceptions, especially regarding polio vaccination in Pakistan [4]. People connect polio vaccines with infertility and are afraid of other consequences [4,10]. Major barriers to vaccination uptake are a lack of appropriate information about these issues due to poor or inadequate communication and a lack of knowledge that negatively affects vaccination rates and awareness [10]. Despite rigorous efforts, immunization coverage in Pakistan has not reached the required level over the last decade. Vaccine confidence plays a vital role in the acceptance and refusal of the vaccination program [12] and improving communication about vaccination can be a crucial factor in enhancing vaccination outcomes [10,13].

Research has identified that community belonging and community influence can be central to behavior change at the individual level [15,16]. Hence external influences such as incentives can act as “cues to action” to motivate caregivers to change their behavior [17,18]. Improved health practices reduced polio vaccine refusals and increased polio vaccine coverage in the target HRUCs, especially for the last remaining resistant populations, to help advance the global agenda of polio eradication.

Global polio eradication hinges on Pakistan’s ability to address the religious, political and socio-economic barriers to immunization, including discrepancies in vaccine coverage, poor health infrastructure and especially inefficient polio worker attitudes and logistical obstacles, particularly the mistrust of cold chain management. Our study aims to evaluate the impact of a novel community engagement and conditional incentives intervention by engaging and empowering community groups. The proposed financial incentive is unique as this initiative would benefit the community as a whole and will be based on the refusal coverage as a whole through community groups. This would help communities to work together and realize the importance of vaccination, and would also help to convince parents who are reluctant, and in turn they would receive a benefit which would be collectively decided by them. This may also foster healthy competition between communities to keep the vaccination coverage up to the mark and receive incentives. The incentives are also conditioned on serial improvements in vaccine coverage; hence, they can counteract any policy resistance. The project intends to create a replicable model that the government can integrate within health systems for long-term sustainability until the goal of eradication of poliovirus is achieved, and would be a cost-effective strategy in the long run due to people’s avoidance of prolonged and repeated campaigns.

## Data Availability

This is a protocol and currently there is no data.
